# Post-Radiotherapy Changes in Circulating Dodecanoic Acid Identify Metabolic Phenotypes Associated with Recurrence in Breast Cancer

**DOI:** 10.3390/biom16030355

**Published:** 2026-02-26

**Authors:** Andrea Jiménez-Franco, Vicente Cambra-Cortés, Raquel García-Pablo, Marta Canela-Capdevila, Rocío Benavides-Villarreal, Xavier Gabaldó-Barrios, Isabel Fort-Gallifa, Jordi Camps, Jorge Joven, Meritxell Arenas

**Affiliations:** 1 Unitat de Recerca Biomèdica, Hospital Universitari de Sant Joan, Institut d’Investigació Sanitària Pere Virgili, Universitat Rovira i Virgili, Av. Dr. Josep Laporte 2, 43204 Reus, Spain; andrea.jimenez@urv.cat (A.J.-F.); vicente.cambra@iispv.cat (V.C.-C.); marta.canela@estudiants.urv.cat (M.C.-C.); jorge.joven@salutsantjoan.cat (J.J.); meritxell.arenas@urv.cat (M.A.); 2 Department of Radiation Oncology, Hospital Universitari de Sant Joan, Institut d’Investigació Sanitària Pere Virgili, Universitat Rovira i Virgili, Av. Dr. Josep Laporte 2, 43204 Reus, Spain; raquel.garciap@estudiants.urv.cat (R.G.-P.); dra.rociobenavides@hotmail.com (R.B.-V.); 3 Department of Clinical Laboratory and Autoimmunity, Infection and Thrombosis Research Group (GRAIIT), Hospital Universitari de Sant Joan, Institut d’Investigació Sanitària Pere Virgili, Universitat Rovira i Virgili, Av. Dr. Josep Laporte 2, 43204 Reus, Spain; xavier.gabaldo@salutsantjoan.cat; 4 Department of Clinical Laboratory, Hospital Universitari Joan XXIII, Institut d’Investigació Sanitària Pere Virgili, Universitat Rovira i Virgili, C. Dr. Mallafré Guasch 4, 43005 Tarragona, Spain; isabel.fort@urv.cat; 5 Oxidative Stress Commission of SemedLab, C. Padilla 323, 08025 Barcelona, Spain

**Keywords:** biomarkers, breast cancer, dodecanoic acid, metabolism, radiotherapy

## Abstract

Research on biomarkers reflecting tumor biology and systemic metabolism is crucial for improving the accuracy and personalization of breast cancer (BC) prognosis. We investigated circulating dodecanoic acid in 229 patients undergoing radiotherapy (RT) and assessed its association with progression-free survival and overall survival over six years. Patients were classified into two phenotypes based on post-RT changes in dodecanoic acid: The Increase Phenotype (IP) had lower baseline concentrations and showed a post-RT rise, whereas the Decrease Phenotype (DP) had higher pre-RT levels and declined after treatment. Dodecanoic acid levels were lower in tumors than in peritumoral samples, and their association with phenotypes varied by sampling region, suggesting that systemic changes reflect broader metabolic adaptations rather than local tissue concentrations. Post-RT increases in dodecanoic acid were associated with higher paraoxonase-1 activity, suggesting a link with antioxidant status. Patients in the IP group had a significantly lower risk of progression than those in the DP group, whereas no significant differences in overall survival were observed. These findings highlight the potential utility of dodecanoic acid measurement as a prognostic biomarker and suggest that modulating fatty acid metabolism could be explored as a therapeutic strategy.

## 1. Introduction

Breast cancer (BC) remains the most diagnosed solid tumor in women and a leading cause of cancer-related mortality worldwide. According to the GLOBOCAN, BC accounted in 2022 for approximately 2.3 million new cases, representing 11.6% of all cancer diagnoses, and close to 666,000 deaths [1]. Recent projections from the International Agency for Research on Cancer indicate that the global burden of this disease is expected to rise substantially in the coming decades, with estimates suggesting that annual cases could reach around 3.2 million, and deaths approximately 1.1 million by 2050 if current trends persist [2]. These forecasts underscore the growing epidemiological weight of BC and highlight the need for improved prevention, early detection, and therapeutic strategies.

The marked heterogeneity of BC continues to demand individualized therapeutic strategies that consider not only patient-specific factors but also tumor stage, molecular subtype, and other indicators of aggressiveness [3]. Current clinical management routinely involves surgery, radiotherapy (RT), chemotherapy, targeted therapies, and endocrine treatments. Neoadjuvant strategies are increasingly employed before surgery to downstage tumors and provide insight into treatment responsiveness, thereby informing subsequent therapeutic decisions [4,5]. Collectively, these interventions aim to eliminate malignant cells, halt disease progression, and improve both survival outcomes and quality of life [6]. This wide heterogeneity in tumor characteristics and treatment responses underscores the need to identify approaches that can better personalize therapy and accurately estimate patient prognosis.

Recent studies have highlighted the pivotal role of metabolism in tumor development and therapeutic response [7,8]. Cancer cells reprogram their metabolic pathways to extract essential substrates even from nutrient-poor environments, thereby supporting their growth, survival, and adaptability [9]. These metabolic adaptations not only influence how tumors respond to therapy but also give rise to distinctive phenotypes that may serve as valuable biomarkers for diagnosis, treatment monitoring, and patient stratification, ultimately enabling more precise and personalized cancer care [10]. In this regard, preliminary studies from our research group suggested that plasma levels of dodecanoic acid could be associated with BC outcomes [11]. This compound, a medium-chain saturated fatty acid (also known as lauric acid), is considered potentially beneficial, as several studies have demonstrated its ability to promote apoptosis and suppress the expression of oncogenic microRNAs in cultured cancer cells [12,13,14]. Moreover, both pharmacological and dietary administration of dodecanoic acid have shown promising results in cancer treatment [15,16,17].

Motivated by these findings, the present study aimed to explore the potential of dodecanoic acid as a prognostic biomarker in BC patients and to investigate its association with long-term clinical outcomes over a 6-year follow-up period.

## 2. Materials and Methods

### 2.1. Study Population and Clinical Design

This prospective study enrolled 229 women diagnosed with invasive BC and treated with curative intent between 2014 and 2022. The median follow-up was 6 years (2–7 years), with some patients reaching 8 years. Patients followed different therapeutic pathways before RT, receiving either surgery or chemotherapy as the initial treatment, based on clinical and molecular characteristics. Eligible participants were ≥18 years of age with histologically confirmed invasive carcinoma. Exclusion criteria included a history of previous oncological disease or Paget’s disease of the nipple. Additionally, individuals with connective tissue or autoimmune disorders (e.g., systemic lupus erythematosus, scleroderma), pregnancy or lactation, severe psychiatric illness, or active infection (including SARS-CoV-2 positivity during the study period) were excluded.

RT was administered to all patients and consisted of hypofractionated whole-breast or chest-wall irradiation (40 Gy in 15 fractions over 3 weeks, 2.67 Gy per fraction). Regional nodal irradiation was added when indicated. A sequential or simultaneous boost to the tumor bed was delivered as needed. Adjuvant chemotherapy and/or hormonal therapy were then given based on tumor molecular subtype, stage, and individual clinical risk.

Progression-free survival (PFS) and overall survival (OS) were evaluated. PFS was defined as the time from study entry to disease progression, including local or regional recurrence, distant metastasis, or death from any cause, whichever occurred first. OS was defined as the time from study entry to death from any cause.

Peripheral blood samples were collected immediately before RT (pre-RT) and again one month after RT completion (post-RT). Each patient’s paired samples were processed under identical conditions for intra-individual comparisons.

Baseline plasma dodecanoic acid levels showed marked inter-individual variability. This heterogeneity motivated exploratory analyses to evaluate whether pre-RT dodecanoic acid concentrations were associated with tumor characteristics. Patients were stratified into Low and High pre-RT groups based on baseline plasma dodecanoic acid levels. The median value of the cohort was used as the cut-off for group assignment. Moreover, plasma dodecanoic acid concentrations exhibited variable responses to RT, increasing in some patients and decreasing in others. Based on these patterns, participants were classified into two groups: the Increase Phenotype (IP), including individuals whose plasma dodecanoic acid levels rose after RT (positive delta: Δ = post-RT − pre-RT > 0), and the Decrease Phenotype (DP), including those whose levels declined after RT (negative delta: Δ = post-RT − pre-RT < 0).

Participants provided written informed consent in line with the ethical guidelines of the 1975 Declaration of Helsinki. The study was approved by the Institutional Review Board (project code: 14/2017) of the Institut d’Investigació Sanitària Pere Virgili. Written informed consent was obtained.

### 2.2. Sample Collection and Processing

Peripheral blood samples were collected to obtain both whole blood and serum for routine laboratory analyses, as well as additional serum and plasma aliquots for the determination of dodecanoic acid levels, oxidative stress, and inflammatory markers. Samples designated for routine analyses were processed immediately, whereas the additional serum and plasma aliquots were stored at −80 °C in our Biobank until batched analyses. Additionally, paired fresh tumor and peritumoral biopsies were obtained in a small subgroup of 11 patients immediately after surgical excision, and were also stored at −80 °C until analytical processing. The sample size for this subgroup was limited because obtaining additional fresh tissue was not feasible due to logistical constraints.

### 2.3. Dodecanoic Acid Measurement

Dodecanoic acid concentrations in plasma and tissue samples were analyzed by chromatographic separation and mass spectrometry.

For plasma samples, protein precipitation was achieved by adding four volumes of methanol containing deuterated myristic acid-d27 (internal standard) to 50 µL of serum. Myristic acid-d27 was used for dodecanoic acid quantification because of its structural similarity to a saturated medium-chain fatty acid and its comparable chromatographic and ionization behavior. Samples were vortexed, incubated at −20 °C for 30 min, and centrifuged at 15,000 rpm at 4 °C. The resulting supernatants were evaporated to dryness and reconstituted in 50 µL of methanol before analysis. Ultra-high-performance liquid chromatography coupled to quadrupole time-of-flight mass spectrometry (UHPLC–qTOF, model 6550, Agilent Technologies, Santa Clara, CA, USA) was used in negative electrospray ionization mode. Chromatographic separation was achieved using a C18 column (ACQUITY UPLC BEH C18, 1.7 µm, 2.1 × 100 mm) and a water–acetonitrile gradient containing 0.05% formic acid. Dodecanoic acid identification was confirmed based on accurate mass, retention time, and tandem mass spectra matched to the Agilent METLIN-PCDL database (>40,000 compounds), and further validated by comparison with pure analytical standards.

For tissue samples, freshly obtained tumor and peritumoral biopsies were homogenized in methanol:water (8:2, *v*/*v*) containing an internal standard using stainless steel beads in a bullet blender. Supernatants were centrifuged (15,000 rpm, 4 °C, 5 min), evaporated at 45 °C, reconstituted in methoxyamine, and incubated for 90 min at 37 °C. Samples were subsequently silylated with N-methyl-N-(trimethylsilyl) trifluoroacetamide containing 1% trimethylchlorosilane for 60 min at room temperature. Gas chromatography coupled to electron ionization and qTOF detection (GC–EI–qTOF, Agilent 7890A coupled to 7200 QTOF) was performed under standard conditions, using helium as the carrier gas (1.1 mL/min). The oven temperature was programmed from 60 °C to 320 °C, and data were acquired in full-scan mode (*m*/*z* 50–600).

Dodecanoic acid was identified using analytical standards and quantified in relative units (RUs), defined as the ratio of the analyte peak area to the internal standard peak area multiplied by 1000, providing a standardized relative measure of compound abundance.

### 2.4. Additional Biomarker and Routine Laboratory Assessments

Plasma levels of interferon-γ (IFN-γ) and serum concentrations of the antioxidant enzyme paraoxonase-1 (PON1) were measured using ELISA kits from Elabscience^®^ (Houston, TX, USA). Serum PON1 activity was assessed by monitoring the hydrolysis rate of phenylacetate at 270 nm in a 9 mM Tris-HCl buffer (pH 8.0) containing 0.9 mM CaCl_2_, as previously described [18]. Plasma concentrations of chemokine (C-C motif) ligand 2 (CCL2) were quantified using ABTS ELISA Development kits (Peprotech, London, UK). Routine clinical parameters, including serum glucose, lipid profile, hepatic enzymes, C-reactive protein, carcinoembryonic antigen (CEA), and cancer antigen 15-3 (CA 15.3), were determined with a COBAS^®^ 8000 automated analyzer (Roche Diagnostics, Basel, Switzerland). Complete blood counts were obtained using a Sysmex XN-1000 automated hematology analyzer (Sysmex, Kobe, Japan).

### 2.5. Statistical Analyses

All statistical analyses were conducted in R (version 4.5.0). Continuous variables are presented as medians and interquartile ranges (IQR, in parentheses). Paired comparisons between pre-RT and post-RT measurements were performed using the Wilcoxon signed-rank test. Between-group comparisons were assessed with the Mann–Whitney U test. Median differences were estimated using the Hodges–Lehmann estimator with corresponding 95% confidence intervals. Spearman’s correlation was used to assess relationships between continuous variables. Associations between Δ-dodecanoic acid (post minus pre-RT) and laboratory or immune markers were evaluated using single linear regression and Spearman correlation analyses. Building on these association tests, baseline biochemical and immune variables were then examined for their capacity to discriminate between metabolic phenotypes. To this end, a LASSO-penalized logistic regression model was fitted using glmnet with cross-validation. Discriminative performance was assessed using receiver operating characteristic (ROC) curves and the area under the curve (AUC).

Cox proportional hazards models were used to evaluate PFS and OS. Hazard ratios (HRs) and 95% confidence intervals (CIs) were estimated. Given the limited number of progression events, multivariable Cox models were parsimoniously adjusted for age only to respect recommended events-per-variable ratios and minimize the risk of model overfitting. A sensitivity analysis was performed to account for potential baseline dependency. Baseline dodecanoic acid levels were incorporated as an additional covariate in the Cox regression model for PFS. All visualizations, including boxplots, scatterplots, heatmaps, forest plots, and ROC curves, were generated using ggplot2, gridExtra, and pROC. All statistical tests were two-sided.

## 3. Results

### 3.1. Demographic, Clinical, and Tumor Characteristics of the Studied Population

The study cohort had a median age of 55 years and was predominantly postmenopausal. Baseline clinical characteristics and comorbidities were well-balanced and are summarized in Table 1.

Most tumors were ductal carcinomas and were diagnosed at early clinical stages (T1–T2), with no nodal involvement and no distant metastases. Histological grade II–III predominated. According to molecular classification, Luminal A and B were the most common subtypes, whereas HER2-positive and triple-negative tumors were less common.

All patients underwent surgical treatment, followed by RT with curative intent. Neoadjuvant chemotherapy was administered in 23.6% of cases, whereas 26.2% of patients received adjuvant chemotherapy. Adjuvant hormone therapy was prescribed in 62.0% of patients, in accordance with hormone receptor status.

During follow-up, disease recurrence was observed in 19 patients (8.3%), including local, regional nodal, and distant metastatic recurrence (6.6%). Among patients with distant recurrence, metastatic sites included bones (*n* = 7), lung (*n* = 2), liver (*n* = 1), and brain (*n* = 1), with several patients presenting metastases in more than one anatomical location (*n* = 7). In addition, 15 patients (6.6%) died during the follow-up period.

### 3.2. Dodecanoic Acid Dynamics Pre- and Post-RT

To investigate whether baseline plasma dodecanoic acid levels were associated with tumor characteristics prior to RT, patients were stratified into Low and High pre-RT groups, with their clinical and tumor features summarized in Table 2. Apart from the expected differences in dodecanoic acid concentrations, no significant differences in baseline clinical or tumor characteristics were observed between the two groups.

**Figure 1:**
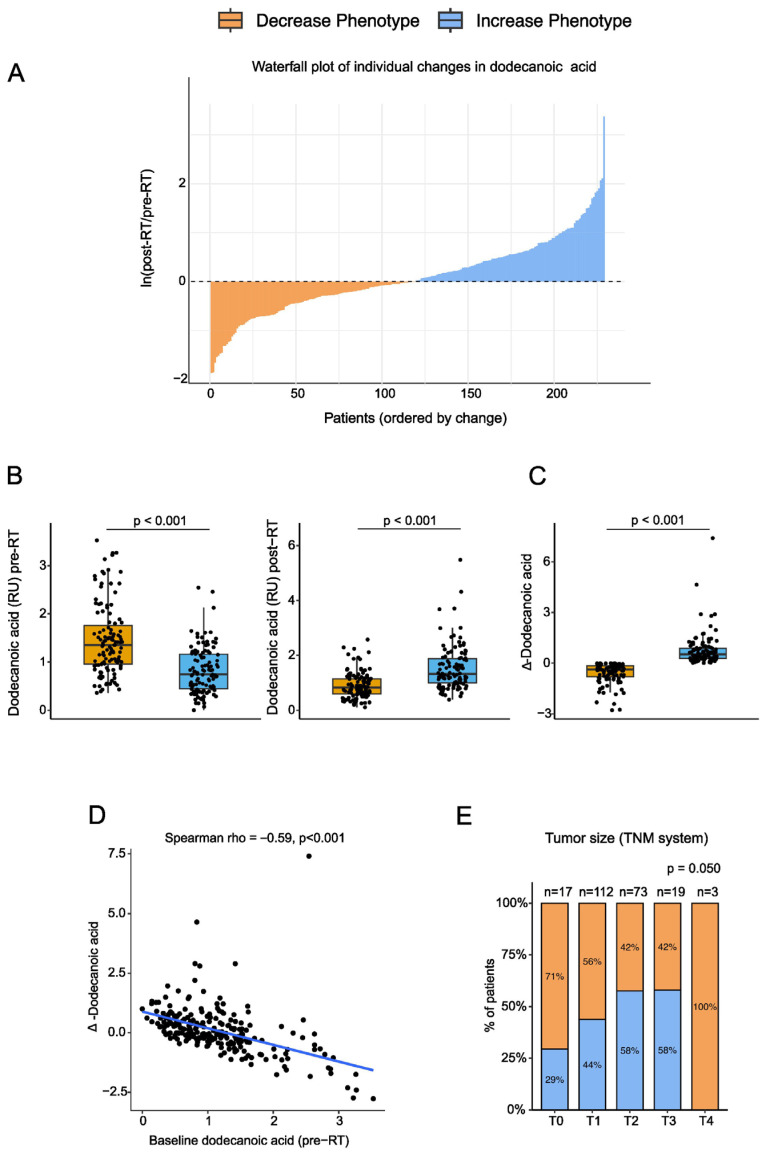
Systemic dodecanoic acid response to radiotherapy: (**A**) Waterfall plot illustrating individual changes in plasma dodecanoic acid, ordered by ln[post-RT/pre-RT] values. Each bar represents one patient, with negative values (to the left of 0) corresponding to the Decrease phenotype and positive values (to the right of 0) corresponding to the Increase phenotype. (**B**,**C**) Boxplots show pre-RT, post-RT, and delta (post–pre) dodecanoic acid levels by phenotype. Individual data points are overlaid (median and interquartile range shown). Two-sided Mann–Whitney tests were used. Phenotype assignment is based on the sign of ln(post-RT/pre-RT). (**D**) Scatterplot showing the association between baseline (pre-RT) dodecanoic acid levels and the post-RT change (Δ = post–pre). (**E**) Distribution of tumor size across phenotypes (Increase vs. Decrease), categorized by TNM stage (T0–T4). Sample sizes include all patients with available paired measurements. Abbreviations: RU, relative units; RT, radiotherapy.

Circulating dodecanoic acid concentrations exhibited substantial inter-individual variability. Among 229 patients, 119 (52.0%) showed a post-RT decline in dodecanoic acid levels (Decrease Phenotype, DP), whereas 110 (48.0%) showed a post-RT increase (Increase Phenotype, IP) (Figure 1A).

Before RT, patients in the DP group had significantly higher plasma dodecanoic acid levels than those in the IP group. After RT, this pattern reversed, with higher post-RT concentrations observed in the IP group. As a result, the net change (Δ-dodecanoic acid) was negative in the DP group and positive in the IP group, confirming a robust divergence between the two metabolic trajectories (Figure 1B,C).

Baseline dodecanoic acid levels were strongly associated with both the magnitude and direction of the post-RT change: patients with lower pre-RT levels tended to exhibit greater increases after treatment, whereas those with higher baseline values frequently showed a decline (*p* < 0.001; Figure 1D).

Most demographic and clinical variables were comparable between phenotypes. Tumor size did not differ significantly between groups; however, a trend was observed across clinical T stages (*p* = 0.050), with the proportion of patients classified as the IP increasing from early (T0–T1) to intermediate stages (T2–T3), while the DP predominated in T0 and T4 tumors (Figure 1E, Supplementary Table S1). No significant differences were observed in molecular subtype, histological grade, or hormone receptor status.

Together, these findings indicate that RT induces patient-specific metabolic shifts in dodecanoic acid, with the direction of the response being strongly influenced by baseline metabolic status and, to a lesser extent, by tumor burden, as discussed further below.

### 3.3. Immune-Metabolic Correlations and Predictive Modeling

We compared pre-RT biochemical and immune markers between groups to assess whether the metabolic phenotypes (IP vs. DP) were associated with baseline characteristics. As shown in Supplementary Table S2, none of the baseline variables reached statistical significance after FDR correction. CEA showed nominal significance in the unadjusted analysis, but this did not persist after multiple testing corrections. All other biochemical and immune parameters, including PON1 concentration, PON1 activity, IFN-γ, and CCL2, were similar between the IP and DP phenotypes, indicating that these phenotypes were not defined by pre-RT systemic profiles.

**Figure 2:**
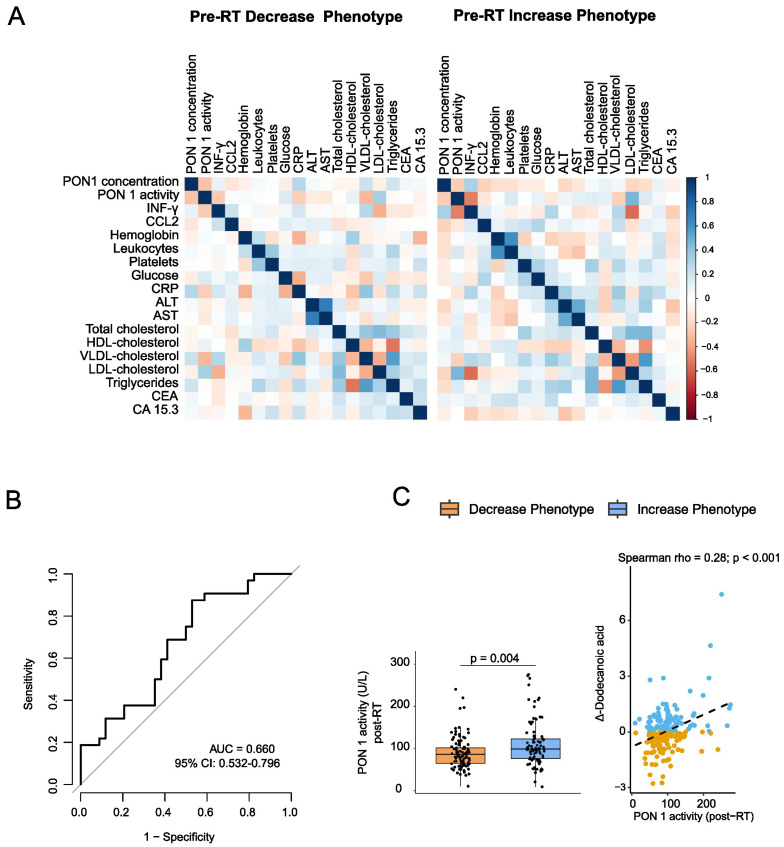
Metabolic and immune correlates of the dodecanoic acid phenotype: (**A**) Baseline immune-metabolic correlation matrices for the Decrease and Increase phenotypes. Spearman correlation coefficients (a measure of monotonic association) were computed using all available pre-radiotherapy (Pre-RT) biochemical and immune markers. Correlation matrices are shown as heatmaps, with blue and red indicating positive and negative correlations, respectively, and color intensity reflecting correlation strength. (**B**) ROC curve for a LASSO-penalized logistic regression model trained on baseline biochemical and immune markers to classify the post-RT metabolic phenotype (Increase vs. Decrease). (**C**) Post-RT PON1 activity according to phenotype (left) and its association with Δ-dodecanoic acid (the change in dodecanoic acid levels after RT (right). Boxplots display the median and interquartile range; *p*-values were obtained using a two-sided Mann–Whitney test. Abbreviations: ALT, alanine aminotransferase; AST, aspartate aminotransferase; AUC, area under the curve; CCL2, chemokine (C-C motif) ligand 2; CEA, carcinoembryonic antigen; CRP, C-reactive protein; HDL, high-density lipoprotein; IFN-γ, interferon-gamma; LDL, low-density lipoprotein; OR, odds ratio; PON1, paraoxonase-1; ROC, receiver operating characteristic; RT, radiotherapy; SD, standard deviation; VLDL, very-low-density lipoprotein.

Because individual markers failed to discriminate between phenotypes, we next explored whether coordinated immune-metabolic patterns might reveal subtle baseline differences. Correlation matrices constructed separately for each phenotype (Figure 2A) revealed largely similar network structures. No phenotype-specific modules or coordinated signatures were identified, and only minor, isolated variations in pairwise correlations were observed. These results suggest that the pre-RT immune-metabolic architecture does not determine which patients will subsequently exhibit the IP or DP phenotype.

To further assess baseline multidimensional differences, we applied LASSO-penalized logistic regression that incorporated all pre-RT biochemical and immune markers. Only two predictors (PON1 activity and leukocyte count) were retained with non-zero coefficients (Supplementary Table S3). Even with this sparse model, discriminatory performance was negligible (ROC AUC = 0.66; Figure 2B), consistent with the lack of meaningful baseline differences in the univariate analyses.

Having confirmed that the metabolic phenotypes were not distinguishable prior to RT, we examined post-treatment immune-metabolic associations. Among all post-RT markers, only PON1 activity showed a significant relationship with Δ-dodecanoic acid (*p* < 0.001; Supplementary Table S4), with higher post-RT PON1 activity associated with greater increases in circulating dodecanoic acid (Figure 2C). Furthermore, PON1 activity was significantly elevated in the IP group compared with the DP group, highlighting its role as the principal immune-metabolic feature associated with the post-RT metabolic response.

### 3.4. Tissue Dodecanoic Acid and Plasma–Tissue Relationships

**Figure 3:**
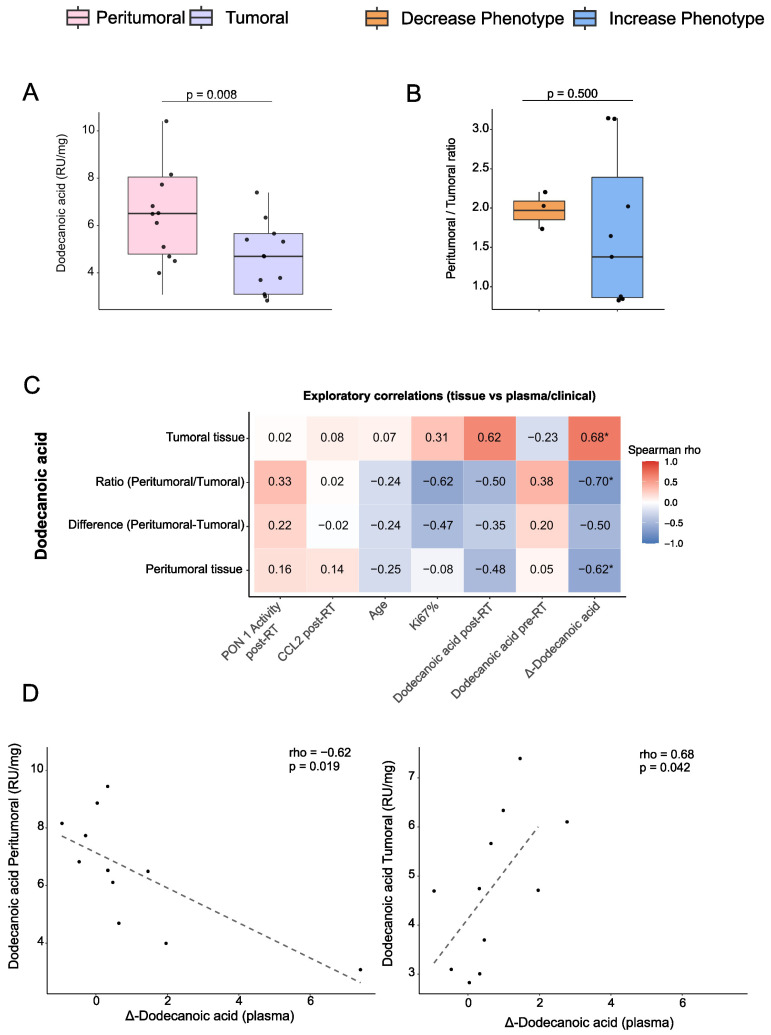
Tissue and systemic dodecanoic acid dynamics: (**A**) Paired comparison of peritumoral and tumoral dodecanoic acid concentrations (*n* = 11 pairs). Boxplots show medians and interquartile ranges (IQRs), with points representing paired observations. Statistical significance was assessed using a paired Wilcoxon signed-rank test. (**B**) Peritumoral-to-tumoral ratio of dodecanoic acid according to plasma phenotype (Increase vs. Decrease). The ratio summarizes the spatial gradient between tissue compartments for each patient. Group comparisons were performed using a two-sided Mann–Whitney test. (**C**) Exploratory Spearman correlation heatmap showing associations between tissue dodecanoic acid metrics and plasma or clinical variables. Correlation matrices are shown as square heatmaps, with blue and red indicating positive and negative correlations, respectively, and color intensity reflecting correlation strength. Tiles display correlation coefficients (ρ); asterisks indicate *p* < 0.05. (**D**) Scatterplots illustrating two key associations: an inverse correlation between peritumoral and plasma Δ-dodecanoic acid, and a positive correlation between tumoral and plasma Δ-dodecanoic acid. Dashed lines indicate linear fits for visualization purposes. These data indicate relative depletion of dodecanoic acid within the tumor microenvironment, with systemic compensatory increases after radiotherapy. Abbreviations: CCL2, chemokine (C-C motif) ligand 2; PON1, paraoxonase-1; RU, relative units; RT, radiotherapy.

To explore whether systemic dodecanoic acid dynamics reflect local changes in the tumor microenvironment, we performed exploratory analyses in a subset of patients with paired tumor and peritumoral biopsies (*n* = 11). Dodecanoic acid levels were higher in peritumoral tissue than in tumor tissue, suggesting relative depletion within the tumor core (Figure 3A). We summarized this gradient for each patient by calculating a peritumoral-to-tumoral ratio based on the spatial analysis. This ratio could be evaluated only in the subset of patients with both tissue compartments and a matched plasma phenotype available. No significant differences were observed between phenotypes; however, this comparison was markedly underpowered due to the limited sample size (Figure 3B).

Correlation analyses revealed contrasting relationships between tissue and plasma dodecanoic acid: peritumoral tissue levels correlated negatively with plasma Δ-dodecanoic acid (*p* = 0.019), whereas tumor tissue levels correlated positively (*p* = 0.042; Figure 3C,D). These patterns suggest that systemic changes may not be directly reflected in tissue concentrations and that the relationship could differ between the tumor core and surrounding tissue. Given the limited sample size, these findings should be interpreted cautiously and considered hypothesis-generating.

### 3.5. Survival Analyses According to Dodecanoic Acid Phenotype

**Figure 4:**
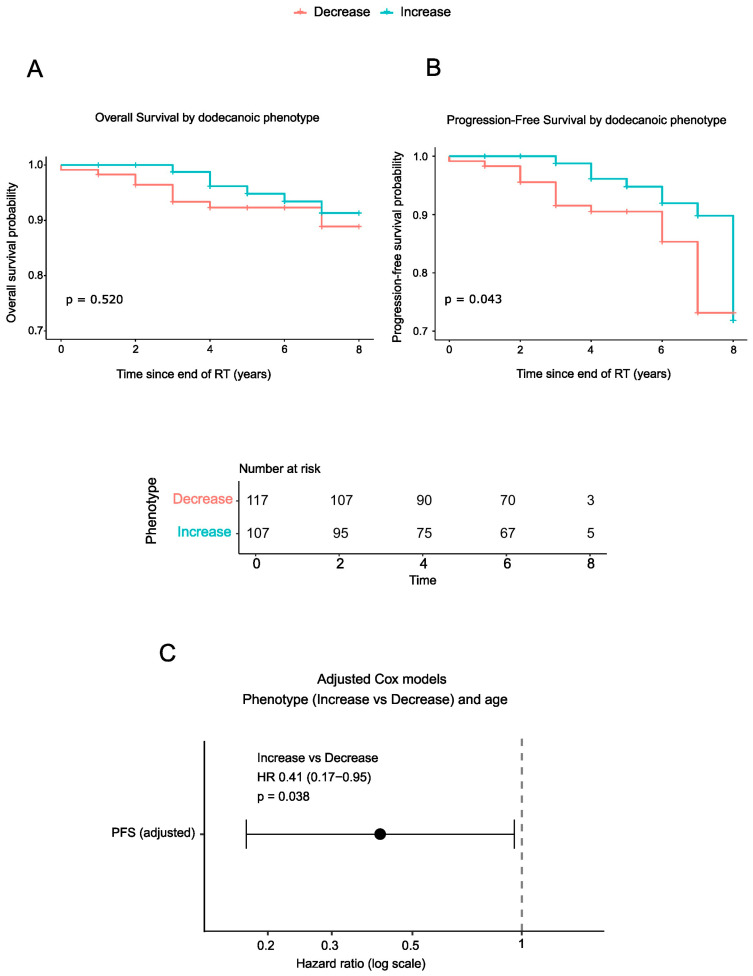
Survival analyses by dodecanoic acid phenotype: (**A**,**B**) Kaplan–Meier curves for OS (**A**) and PFS (**B**) by dodecanoic acid phenotype (Decrease vs. Increase). Numbers at risk are displayed in the PFS panel; they are identical for both outcomes. Two-sided log-rank *p*-values are indicated on the panels. (**C**) Adjusted Cox proportional hazards regression model for PFS according to dodecanoic acid phenotype (Increase vs. Decrease) adjusted for age. Hazard ratios (HRs) and 95% confidence intervals (CIs) are shown on a logarithmic scale; HR < 1 indicates a lower risk of disease progression for the Increase phenotype. Abbreviations: CI, confidence interval; HR, hazard ratio; OS, overall survival; PFS, progression-free survival; RT, radiotherapy.

To investigate the potential prognostic significance of systemic dodecanoic acid modulation after RT, we analyzed PFS and OS according to plasma phenotype. Kaplan–Meier analysis revealed no significant differences in OS between dodecanoic acid phenotypes. However, patients with increased dodecanoic acid levels after RT showed a significantly improved PFS compared with those in the DP group (log-rank *p* = 0.043; Figure 4A,B).

Building on these Kaplan–Meier results, Cox proportional hazards regression analysis demonstrated that the IP was independently associated with a reduced risk of disease progression (HR = 0.41, 95% CI: 0.17–0.95; *p* = 0.038) after adjustment for age (Figure 4C, Table 3). Given the strong inverse association observed between baseline dodecanoic acid levels and post-RT changes, a sensitivity analysis was performed, including baseline dodecanoic acid levels as an additional covariate in the Cox regression model. The association between the post-RT dodecanoic acid phenotype and PFS remained statistically significant after this adjustment, supporting the robustness of the observed prognostic relationship (Supplementary Table S5).

## 4. Discussion

The most relevant finding of this prospective study is the identification of two distinct systemic metabolic phenotypes based on post-RT changes in circulating dodecanoic acid (IP and DP) and the observation that patients in the IP group exhibited improved PFS over a 6-year follow-up. It is important to emphasize that these findings should be interpreted as associative rather than causal. The observed metabolic trajectories are more likely to reflect underlying differences in tumor biology, treatment sensitivity, or host metabolic resilience than a direct antitumor effect of dodecanoic acid itself.

A possible interpretation of the post-RT increase in circulating dodecanoic acid is that it may reflect a favorable systemic metabolic adaptation. Patients in the IP group had lower pre-RT dodecanoic acid levels, suggesting efficient mitochondrial β-oxidation and greater metabolic flexibility [19,20]. Following RT, tumor cell damage may likely reduce dodecanoic acid consumption, leading to higher circulating levels in these patients [6,9]. This increase may represent a combination of reduced tumor demand, restored systemic metabolic homeostasis, and improved antioxidant and inflammatory balance, as suggested by higher PON1 activity [21,22]. In contrast, patients in the DP group had higher pre-RT dodecanoic acid, possibly reflecting impaired mitochondrial utilization or metabolic inflexibility [23]. After RT, these patients may experience further metabolic stress or a diversion of fatty acids toward inflammatory pathways, leading to decreased circulating dodecanoic acid and a less favorable prognosis. The strong inverse association between baseline dodecanoic acid levels and post-RT changes suggests that regression-to-the-mean effects may partially contribute to the observed phenotypic divergence. However, in sensitivity analyses adjusting for baseline dodecanoic acid levels, the association between post-RT phenotype and PFS remained statistically significant. This finding argues against regression-to-the-mean as the sole explanation for the observed prognostic relationship, although confirmation in an independent cohort will be necessary. Taken together, our results reinforce the prognostic relevance of post-RT changes in circulating dodecanoic acid, while acknowledging that they do not demonstrate a causal or therapeutic effect. The divergence between plasma and tissue dodecanoic acid provides further insight into systemic metabolic adaptations. Tumor tissue exhibited lower concentrations than peritumoral tissue, and tissue measurements showed distinct correlation patterns with plasma Δ-dodecanoic acid. If systemic changes simply mirrored local tumor concentrations, plasma levels would be expected to correlate uniformly with tumor tissue. Instead, the observed differential associations suggest that circulating dodecanoic acid dynamics are influenced by broader systemic processes, rather than solely by local tumor uptake. Lower tumor tissue levels likely reflect basal consumption by cancer cells to support growth and survival, as previously reported [23,24,25]. Importantly, higher circulating dodecanoic acid levels may serve as a surrogate marker of systemic metabolic recovery after RT, rather than acting as a direct mediator of tumor control [26]. While experimental studies provide valuable biological context, the present clinical data do not allow causal inference and should not be interpreted as evidence of a direct antitumor mechanism in patients.

Integrating these observations with the phenotypic patterns, IP patients appear to display a systemic metabolic profile that recovers more effectively after treatment. This pattern of metabolic recovery, reflected by post-RT increase in circulating dodecanoic acid, may be associated with lower risk of disease recurrence, consistent with the improved PFS observed in this group [27]. Monitoring post-RT dynamics of dodecanoic acid could therefore provide insight into both systemic metabolic recovery and tumor vulnerability, offering a novel perspective on prognostic biomarkers in BC. Although recurrence was evaluated as a clinical endpoint in this study, metastatic dissemination represents a dynamic biological process shaped by systemic adaptation and intercellular communication. The tumor microenvironment in BC includes extracellular vesicles that mediate the transfer of proteins, nucleic acids, and lipids, contributing to tumor progression and metastatic conditioning. While the present study did not assess vesicle-associated lipid transport, systemic shifts in circulating fatty acids following RT may occur within broader networks of intercellular metabolic signaling that influence tumor-host interactions. In this context, post-RT changes in circulating dodecanoic acid may reflect systemic vulnerability or resistance rather than an isolated metabolic fluctuation [28].

An additional biological axis that may integrate these metabolic and immune adaptations is hypoxia-driven signaling. Hypoxia-driven signaling pathways may provide an additional biological bridge linking metabolic adaptation and immune regulation in the post-RT setting. RT can induce transient hypoxic stress within tumor tissues, activating hypoxia-responsive transcriptional programs that coordinate mitochondrial function, lipid metabolism, and immune modulation. Hypoxia-associated molecular mediators, such as the BCL2-interacting protein 3, have been implicated in the interplay between mitophagy, metabolic reprogramming, and immune-related pathways in BC. Although hypoxia markers were not directly evaluated in the present study, alterations in circulating dodecanoic acid dynamics may reflect systemic adaptations occurring within hypoxia-responsive metabolic networks. Within this framework, metabolic phenotypes observed after RT could be interpreted as part of a broader hypoxia–metabolism–immunity axis influencing disease progression, rather than as isolated biochemical fluctuations [29].

Beyond mechanistic insights, our findings have potential clinical relevance. We observed that post-RT increases in circulating dodecanoic acid are associated with lower progression risk. Rather than positioning plasma dodecanoic acid as a standalone predictor, these results suggest that dynamic metabolic changes following RT may contribute to a broader prognostic framework. Circulating metabolic markers are increasingly understood as components of multilayered biomarker ecosystems that integrate metabolic, immunological, and clinicopathological dimensions within precision oncology. In this context, post-RT dodecanoic acid dynamics could complement established parameters, such as tumor size, stage, and molecular subtype, providing additional systemic information for recurrence risk assessment [30]. Assessing dodecanoic acid dynamics after RT may help stratify patients by progression risk. This can support closer monitoring of those with unfavorable profiles and potentially inform post-treatment surveillance or adjuvant therapeutic decisions. Moreover, as dodecanoic acid has demonstrated antitumor effects in preclinical models [12,13,14], the present findings do not support therapeutic modulation. Rather, they suggest that post-RT dodecanoic acid dynamics may warrant further investigation as a prognostic biomarker in further prospective and interventional studies. Overall, our study highlights the importance of integrating metabolic biomarkers into clinical decision-making, opening avenues for more personalized BC care.

## 5. Strengths and Limitations

Strengths of our study include its prospective design, long-term follow-up of up to six years, and comprehensive integration of metabolomic, immunologic, and clinical data. By combining systemic metabolic profiling with standard clinicopathological assessments, our findings provide a robust framework for developing more personalized prognostic tools and may help inform tailored therapeutic strategies.

Despite these strengths, several limitations should be acknowledged. The study was conducted at a single center, and analysis of paired tumor and peritumoral tissue samples was limited to a small subset of patients (*n* = 11), thereby substantially reducing statistical power and rendering the findings exploratory. A further limitation of this study is the limited number of progression events, which restricted the inclusion of multiple clinicopathological covariates in the survival models. Although tumor stage, nodal status, molecular subtype and systemic treatments are well-established prognostic factors in BC, adjustment for these variables was not feasible without substantially increasing the risk of model overfitting. Therefore, residual confounding factors cannot be excluded and should be addressed in future studies with larger event numbers. Validation in independent, larger cohorts is therefore necessary to confirm the prognostic value of dodecanoic acid phenotypes and further elucidate the underlying biological mechanisms.

## 6. Conclusions

Post-RT increases in circulating dodecanoic acid were associated with a reduced progression risk in patients with BC. Inter-individual variability in baseline dodecanoic acid levels was linked to distinct metabolic responses to RT, supporting the potential prognostic relevance of this metabolite. Future studies are warranted to validate these findings in independent cohorts, elucidate the mechanistic links between systemic metabolism and tumor biology, and explore whether modulation of dodecanoic acid-related pathways may improve treatment outcomes.

## Figures and Tables

**Table 1:** Demographic and clinical characteristics of included patients.

Clinical Characteristics	
Age at diagnosis (years)	55 (48–64)
Smoking habit	50 (21.8)
Alcohol habit (>20 g/day)	15 (6.6)
Diabetes mellitus	15 (6.6)
Hypertension	52 (22.7)
Dyslipidemia	53 (23.1)
Chronic obstructive pulmonary disease	10 (4.4)
Ischemic heart disease	7 (3.1)
Menopause status	
Premenopausal	52 (22.7)
Peri-menopausal	27 (11.8)
Postmenopausal	145 (63.3)
No data	5 (2.2)
Use of oral contraceptives	73 (31.9)
Motherhood	172 (75.1)
Family history of cancer	101 (44.1)
**Cancer Characteristics**	
Tumor size (TNM system)	
T0	17 (7.4)
T1	113 (49.3)
T2	72 (31.4)
T3	19 (8.3)
T4	3 (1.3)
No data	5 (2.2)
Nodes (TNM system)	
N0	149 (65.1)
N1	54 (23.6)
N2	17 (7.4)
N3	4 (1.7)
No data	5 (2.2)
Metastases (TNM system)	
M0	229 (100)
M1	-
Pathological anatomy	
Ductal carcinoma	190 (83.8)
Lobular carcinoma	8 (3.9)
Other	23 (10.0)
No data	6 (2.6)
Histological grade	
I	44 (19.2)
II	118 (51.5)
III	62 (27.1)
No data	5 (2.2)
Estrogen receptors	
Positive	184 (80.3)
Negative	40 (17.5)
No data	5 (2.2)
Progesterone receptors	
Positive	149 (51.1)
Negative	75 (32.8)
No data	5 (2.2)
HER2-positive	41 (17.9)
Ki67 antigen	
0%	1 (0.4)
1–15%	81 (35.4)
16–50%	109 (47.6)
>50%	33 (14.4)
No data	5 (2.2)
Tumor molecular classification	
Luminal A	64 (27.9)
Luminal B	89 (38.9)
HER2-positive	40 (17.5)
Triple negative	31 (13.5)
No data	5 (2.2)
**Oncological treatments**	
Surgery	229 (100)
Neoadjuvant chemotherapy	54 (23.6)
Adjuvant chemotherapy	60 (26.2)
Adjuvant hormone therapy	142 (62.0)
Adjuvant radiotherapy	220 (100)
**Follow up**	
Follow up (years)	6 (2–7)
Cancer Recurrence	
Local	3 (1.3)
Regional (nodal)	1 (0.4)
Distant (metastatic)	15 (6.6)
No data	5 (2.2)
Deceased	15 (6.6)

Values are expressed as *n* (percentage) or median (interquartile range). Abbreviations: HER2, human epidermal growth factor receptor 2; M0/1, absence/presence of distant metastasis; N0–3 lymph node status; T0–4, tumor size.

**Table 2:** Baseline tumor characteristics stratified by pre-radiotherapy dodecanoic acid levels.

	Low Pre-RT (*n* = 115)	High Pre-RT (*n* = 114)	*p*-Value	FDR
Pre-RT dodecanoic acid (RU)	0.65 (0.43–0.84)	1.47 (1.24–1.98)	4.53 × 10^−39^	4.53 × 10^−38^
Age at diagnosis (years)	58 (48.5–65.5)	53.5 (47–62)	0.035	0.175
Tumor size (TNM system)			0.270	0.338
T0	5 (4.5)	12 (10.7)		
T1	56 (50.0)	57 (50.9)		
T2	41 (36.6)	31 (27.7)		
T3	8 (7.1)	11 (9.8)		
T4	2 (1.8)	1 (0.9)		
No data	2 (1.8)	2 (1.8)		
Nodes (TNM system)			0.110	0.276
N0	74 (66.1)	76 (67.9)		
N1	31 (27.7)	22 (19.6)		
N2	7 (6.2)	10 (8.9)		
N3	-	4 (3.6)		
No data	2 (1.8)	2 (1.8)		
Histological grade			0.721	0.721
I	21 (18.8)	23 (20.5)		
II	62 (55.4)	56 (50.0)		
III	29 (25.9)	33 (29.5)		
No data	3 (2.6)	2 (1.75)		
Estrogen receptors			0.600	0.667
Positive	90 (89.4)	94 (83.9)		
Negative	22 (19.6)	18 (16.1)		
No data	3 (2.6)	2 (1.75)		
Progesterone receptors			0.257	0.337
Positive	70 (62.5)	79 (70.5)		
Negative	42 (37.5)	33 (29.5)		
No data	3 (2.6)	2 (1.75)		
HER2-positive	17 (15.2)	25 (22.3)	0.231	0.337
Ki67 antigen			0.196	0.337
0%	34 (30.4)	47 (42.0)		
1–15%	60 (53.6)	49 (43.8)		
16–50%	17 (15.2)	16 (14.3)		
>50%	1 (0.9)	-		
No data	3 (2.6)	2 (1.8)		
Tumor molecular classification			0.062	0.207
Luminal A	26 (23.2)	38 (33.9)		
Luminal B	51 (45.5	38 (33.9)		
HER2-positive	16 (14.3)	24 (21.4)		
Triple negative	19 (17.0)	12 (10.7)		
No data	3 (2.6)	2 (1.8)		

Values are presented as *n* (percentage) or median (interquartile range). Statistical comparisons were performed using the Mann–Whitney U test for continuous variables and adjusted for multiple comparisons using the Benjamini–Hochberg false discovery rate (FDR) method. Fisher’s exact test was used for categorical variables. Abbreviations: HER2, human epidermal growth factor receptor 2; M0/1, absence/presence of distant metastasis; N0–3 lymph node status; RU: relative units; RT: radiotherapy; T0–4, tumor size.

**Table 3:** Multivariable Cox regression analyses for progression-free survival (PFS).

Outcome	Variable	HR	95% CI	*p*-Value
PFS	Phenotype (increase/decrease)	0.41	0.17–0.95	0.038
	Age at diagnosis (year)	0.98	0.95–1.02	0.350

PFS was defined as the time from the end of radiotherapy to disease recurrence, distant metastasis, or death, whichever occurred first. Hazard ratios (HRs) and 95% confidence intervals (CIs) are shown. The multivariable model was adjusted for age. HR < 1 indicates a lower risk of progression. The dodecanoic acid phenotype was defined based on the direction of change after radiotherapy.

## Data Availability

The data presented in this study are available on request from the corresponding author due to privacy and confidentiality reasons.

## Supplementary Materials

The following supporting information can be downloaded at: https://www.mdpi.com/article/10.3390/biom16030355/s1, Table S1: Clinical and tumor characteristics of breast cancer patients according to dodecanoic acid response to radiotherapy; Table S2: Pre-radiotherapy biochemical and immune markers according to dodecanoic acid response phenotype; Table S3: Coefficients from the LASSO-penalized logistic regression model (pre-radiotherapy variables); Table S4: Association between biochemical and immune markers and Δ-dodecanoic acid (post-pre) measured post-radiotherapy. Table S5: Sensitive Cox proportional hazards model for progression-free survival adjusted for age and baseline dodecanoic acid levels.

## Abbreviations

The following abbreviations are used in this manuscript:

AUC	Area under the curve
BC	Breast cancer
CA 15.3	Cancer antigen 15.3
CCL2	Chemokine (C-C motif) ligand 2
CEA	Carcinoembryogenic antigen
CRP	C-reactive protein
DP	Decrease phenotype
FDR	False discovery rate
HDL	High-density lipoprotein
HR	Hazard ratio
IFN-γ	Interferon-γ
IP	Increase phenotype
IQR	Interquartile range
LDL	low-density lipoprotein
OR	Odds ratio
OS	Overall survival
PFS	Progression-free survival
PON1	Paraoxonase-1
qTOF	Quadrupole time-of-flight
ROC	Receiver operating characteristic
RT	Radiotherapy
SD	Standard deviation
UHPLC	Ultra-high-performance liquid chromatography
VLDL	Very-low-density lipoprotein
