# Supplementary material for: Post-Radiotherapy Changes in Circulating Dodecanoic Acid Identify Metabolic Phenotypes Associated with Recurrence in Breast Cancer
Source: Biomolecules. 2026 Feb 26;16(3):355. doi: 10.3390/biom16030355 (PMC13023575; doi:10.3390/biom16030355)
Supplement: Supplementary file 1 [file biomolecules-16-00355-s001.zip › biomolecules-4148387-supplementary.pdf]

Table S1. Clinical and tumor characteristics of breast cancer patients according to dodecanoic acid response to radiotherapy.

	Decrease Phenotype (n = 119)	Increase Phenotype (n = 110)	p-value
Dodecanoic acid pre-RT	1.4 (1.0-1.8)	0.7 (0.4-1.2)	**<0.001**
Dodecanoic acid post-RT	0.8 (0.6-1.1)	1.3 (1.0-1.9)	**<0.001**
Delta dodecanoic acid (ln(postRT-preRT))	-0.4 (-0.8,-0.2)	0.5 (0.3-0.9)	**<0.001**
Clinical Characteristics			
Age at diagnosis (years)	55 (49-64)	57 (47-65)	0.967
Smoking habit	25 (21.0)	25 (22.7)	0.825
Alcohol habit (>20g/day)	7 (5.9)	8 (7.3)	0.629
Diabetes mellitus	10 (8.4)	5 (4.5)	0.459
Hypertension	30 (25.2)	22 (20.0)	0.583
Dyslipidemia	27 (22.7)	26 (23.6)	0.927
Chronic obstructive pulmonary disease	4 (3.4)	6 (5.5)	0.718
Ischemic heart disease	4 (3.4)	3 (2.7)	0.923
Menopause status			0.790
Premenopausal	24 (20.2)	28 (25.5)	
Peri-menopausal	15 (12.6)	12 (10.9)	
Postmenopausal	77 (64.7)	68 (61.8)	
No data	3 (2.5)	2 (1.8)	
Use of oral contraceptives	37 (31.1)	36 (32.7)	0.273
Motherhood	88 (73.9)	84 (76.4)	0.111
Family history of cancer	64 (53.8)	63 (57.3)	0.426
Cancer Characteristics			
Tumor size (TNM system)			0.050
T0	12 (10.1)	5 (4.5)	
T1	62 (52.1)	50 (45.5)	
T2	31 (26.1)	42 (38.2)	
T3	8 (6.7)	11 (10.0)	
T4	3 (2.5)	-	
No data	3 (2.5)	2 (1.8)	
Nodes (TNM system)			0.474
N0	80 (67.2)	69 (62.7)	
N1	23 (19.3)	31 (28.2)	
N2	10 (8.4)	7 (63.4)	
N3	3 (2.5)	1 (0.9)	
No data	3 (2.5)	2 (1.8)	
Metastases (TNM system)			1.000
M0	119 (100)	110 (100)	
M1	-	-	
Pathological anatomy of the tumor			0.296
Ductal carcinoma	103 (86.6)	89 (80.9)	
Lobular carcinoma	2 (1.7)	7 (6.4)	
Other	11 (9.2)	12 (10.9)	
No data	3 (2.5)	2 (1.8)	
Histological grade			0.628
I	23 (19.3)	21 (19.1)	

II	65 (54.6)	53 (48.2)	
III	28 (23.5)	34 (30.9)	
No data	3 (2.5)	2 (1.8)	
Estrogen receptors			0.454
Positive	92 (77.3)	91 (82.7)	
Negative	24 (20.2)	17 (15.5)	
No data	3 (2.5)	2 (1.8)	
Progesterone receptor			0.152
Positive	71 (59.7)	74 (67.3)	
Negative	45 (37.8)	34 (30.9)	
No data	3 (2.5)	2 (1.8)	
HER2 positive	22 (18.5)	20 (18.2)	0.904
Ki67 antigen			0.505
0%	-	1 (0.9)	
1-15%	42 (35.3)	39 (35.5)	
16-50%	53 (44.5)	56 (50.9)	
>50%	21 (17.6)	12 (10.9)	
No data	3 (2.5)	2 (1.8)	
Tumor molecular classification			0.813
Luminal A	30 (25.2)	34 (30.9)	
Luminal B	47 (39.5)	43 (39.1)	
HER 2 positive	21 (17.6)	19 (17.3)	
Triple negative	18 (15.1)	12 (10.9)	
No data	3 (2.5)	2 (1.8)	
Oncological treatment			
Surgery	119 (100)	110 (100)	
Neoadjuvant chemotherapy	32 (25.2)	22 (20)	
Adjuvant chemotherapy	28 (23.5)	32 (29.1)	
Adjuvant hormone therapy	70 (58.8)	72 (65.5)	
Adjuvant radiotherapy	119 (100)	110 (100)	
Follow up			
Cancer recurrence			0.296
Local	2 (1.7)	1 (0.9)	
Regional (nodal)	1 (0.8)	-	
Distant (metastatic)	10 (8.4)	5 (4.5)	
No data	3 (2.5)	2 (1.8)	
Deceased	9 (7.6)	6 (5.5)	0.753

Values are presented as n (percentage) or median (interquartile range). Statistical comparisons were performed using the Mann-Whitney U test for continuous variables and Fisher's exact test for categorical variables. Bold values denote statistically significant differences ($p<0.05$). Abbreviations: BC, breast cancer; HER2, human epidermal growth factor receptor 2; M0/1, absence/presence of distant metastasis; N0-3 lymph node status; RT, radiotherapy; T0-4, tumor size.

Table S2. Pre-radiotherapy biochemical and immune markers according to dodecanoic acid response phenotype.

	Decrease Phenotype (n = 119)	Increase Phenotype (n = 110)	p-value	FDR
CEA (ng/mL)	2.1 (1.5-2.8)	2.8 (1.9-3.9)	0.008	0.143
CA 15.3 (U/mL)	16.5 (12.6-22.3)	18.6 (14.8-25.8)	0.067	0.482
Glucose (mg/dL)	92.5 (86.8-103.0)	95.5 (89.0-111.0)	0.104	0.482
INF-γ (pg/mL)	238.5 (151.1-374.7)	303.3 (173.1-633.7)	0.108	0.482
PON1 concentration (mg/L)	84.5 (41.4-116.8)	65.0 (23.2-113.4)	0.145	0.482
PON1 activity (U/mL)	99.4 (78.0-122.6)	103.4 (87.3-122.7)	0.180	0.482
Total cholesterol (mmol/L)	5.6 (4.7-6.2)	5.7 (5.0-6.3)	0.205	0.482
ALT (U/L)	0.3 (0.2-0.5)	0.3 (0.2-0.4)	0.214	0.482
CCL2 (pg/mL)	53.7 (34.9-70.7)	60.2 (33.9-79.7)	0.306	0.587
VLDL-cholesterol (mmol/L)	0.7 (0.5-1.6)	0.8 (0.5-2.4)	0.342	0.588
CRP (mg/dL)	0.3 (0.2-0.5)	0.3 (0.2-0.6)	0.413	0.587
Platelets (x10^9/L)	250.0 (208.5-290.5)	215.0 (215.0-305.0)	0.425	0.587
Triglycerides (mmol/L)	1.3 (0.9-1.9)	1.3 (1.0-1.8)	0.427	0.587
Hemoglobin (g/dL)	12.8 (11.8-13.6)	12.8 (11.9-13.8)	0.457	0.587
AST (U/L)	0.3 (0.3-0.4)	0.3 (0.3-0.4)	0.527	0.874
LDL-cholesterol (mmol/L)	2.7 (1.7-3.5)	2.8 (1.2-3.6)	0.953	0.988
Leukocytes (x10^9/L)	5.5 (4.4-7.0)	5.7 (4.1-7.8)	0.981	0.988
HDL-cholesterol (mmol/L)	1.6 (1.3-1.9)	1.6 (1.4-1.8)	0.981	0.988

Values are expressed as median (interquartile range) of pre-RT measurements. p-values were calculated using the Wilcoxon test and adjusted for multiple comparisons using the Benjamini–Hochberg false discovery rate (FDR) method. Variables are ranked by ascending p-value, and only the most relevant biochemical and immune parameters are displayed.

Abbreviations: ALT, alanine aminotransferase; AST, aspartate aminotransferase; CA15.3, cancer antigen 15.3; CCL2, monocyte chemoattractant protein-1; CEA, carcinoembryonic antigen; CRP, C-reactive protein; HDL, high-density lipoprotein cholesterol; IFN-γ, interferon-gamma; LDL, low-density lipoprotein cholesterol; PON1, paraoxonase 1.

Table S3. Coefficients from the LASSO-penalized logistic regression model (pre-radiotherapy variables).

Variable	Coefficient (λmin)	OR*	Selected by model
PON1 activity	0.000834	1.00	Yes
Leukocytes	-0.0514	0.95	Yes
PON1 concentration	0	1	No
INF- γ	0	1	No
CCL2	0	1	No
Hemoglobin	0	1	No
Platelets	0	1	No
Glucose	0	1	No
CRP	0	1	No
AST	0	1	No
ALT	0	1	No
GOT	0	1	No
Total cholesterol	0	1	No
HDL-cholesterol	0	1	No
VLDL-cholesterol	0	1	No
LDL-cholesterol	0	1	No
Triglycerides	0	1	No
CEA	0	1	No
CA 15.3	0	1	No

*OR approximate, based on standardized predictors (glmnet default scaling).

Values correspond to standardized coefficient obtained from the LASSO-penalized logistic regression model including all baseline biochemical and immune variables as candidate predictors. Predictors with a coefficient of zero were removed from the model by the penalization procedure. Odds ratios (OR) are approximate and represent the change in odds of belonging to the Increase Phenotype per one standard deviation increase in each marker. Only predictors with non-zero coefficients contributed to the final model.

Abbreviations: ALT, alanine aminotransferase; AST, aspartate aminotransferase; CA15.3, cancer antigen 15.3; CCL2, monocyte chemoattractant protein-1; CEA, carcinoembryonic antigen; CRP, C-reactive protein; HDL, high-density lipoprotein cholesterol; IFN-γ, interferon-gamma; LDL, low-density lipoprotein cholesterol; PON1, paraoxonase 1.

Table S4. Association between biochemical and immune markers and Δ-dodecanoic acid (post-pre) measured post-radiotherapy.

	Spearman (rho)	p-value (Spearman)	β (linear model)	p-value (linear)	FDR
PON1 activity	0.3	<0.001	0.009	**<0.001**	**<0.001**
PON1 concentration	-0.1	0.045	-0.002	**0.037**	0.334
CEA	0.2	0.034	0.08	0.107	0.642
CA 15.3	0.07	0.503	0.02	0.371	0.730
INF-γ	0.04	0.637	0.0001	0.445	0.730
CCL2	0.02	0.741	0.0003	0.564	0.730
Total cholesterol	0.06	0.371	0.04	0.557	0.730
HDL-cholesterol	-0.02	0.775	-0.2	0.290	0.730
VLDL-cholesterol	0.1	0.151	0.07	0.195	0.730
LDL-cholesterol	-0.07	0.303	-0.03	0.564	0.730
Triglycerides	0.08	0.238	0.1	0.254	0.730
CRP	-0.1	0.061	-0.03	0.539	0.730
AST	-0.01	0.881	0.2	0.608	0.730
Leukocytes	0.1	0.235	0.03	0.576	0.730
Plateles	0.07	0.438	0.002	0.325	0.730
Glucose	0.05	0.737	0.002	0.812	0.914
Hemoglobin	0.08	0.339	-2.3×10^{-06}	0.880	0.932
ALT	-0.06	0.377	0.02	0.949	0.949

Δ-dodecanoic acid was calculated as the difference between post-radiotherapy (post-RT) and pre-radiotherapy (pre-RT) plasma concentrations. Associations between Δ-dodecanoic acid and each biochemical or immune marker (measured post-RT) were evaluated using Spearman correlation and simple linear regression. Reported p-values correspond to the linear regression models, and the false discovery rate (FDR) was controlled using the Benjamini–Hochberg procedure. Bold values denote statistically significant differences ($p<0.05$).

Abbreviations: ALT, alanine aminotransferase; AST, aspartate aminotransferase; CA15.3, cancer antigen 15.3; CCL2, monocyte chemoattractant protein-1; CEA, carcinoembryonic antigen; CRP, C-reactive protein; HDL, high-density lipoprotein cholesterol; IFN-γ, interferon-gamma; LDL, low-density lipoprotein cholesterol; PON1, paraoxonase 1.

Table S5. Sensitive Cox proportional hazards model for progression-free survival adjusted for age and baseline dodecanoic acid levels.

	HR	95% CI	p-value
Increase vs Decrease phenotype	0.30	0.12-0.75	0.010
Age (per year)	0.98	0.95-1.01	0.268
Baseline dodecanoic acid	0.57	0.28-1.16	0.119

Multivariate Cox proportional hazards model evaluating PFS, including phenotype (Increase vs. Decrease), age at diagnosis, and baseline dodecanoic acid levels (pre-RT). Hazard ratios are presented with 95% confidence intervals.

Abbreviations: CI, confidence interval; HR hazard ratio; PFS, progression-free survival; RT, radiotherapy.
